# Serving Up Support: Understanding the Needs and Experiences of Sandwich Caregivers

**DOI:** 10.1093/geroni/igaf122.2158

**Published:** 2025-12-31

**Authors:** Autumn Decker, Raven Weaver, Nancy Swanger, Darcie Bagott

**Affiliations:** Rede Group, Pullman, Washington, United States; Washington State University, Pullman, Washington, United States; Washington State University, Pullman, Washington, United States; Washington State University, Pullman, Washington, United States

## Abstract

In the United States, there are an estimated 2.5 million adults living as “sandwich caregivers,” or people who are simultaneously caring for aging parents and children. These caregivers face similar struggles and difficulties to other family caregivers (e.g., high levels of stress, burnout), but report even higher levels of financial and emotional difficulty, yet less is known about the unique hopes, fears, and needs of these carers. Using Atlas.ti software, we conducted inductive content analysis of the 30 top posts each month, posts that had the highest engagement including views, comments, and reactions, in a Facebook support group for sandwich caregivers from the past 6 months (August 2024-February 2025; we anticipate updating results through August 2025). This evolving group (n = 32,633) includes members from 91 different countries who are majority Women (96%) and those between the ages of 45-54 (45%) and 55-64 (32%). Analysis of 180 monthly posts revealed several themes including needing instrumental advice, seeking emotional support, or wanting help navigating family relationships and priorities. Common topics of posts included end-of-life care needs, grief, financial, physical, and mental health concerns, and healthcare and insurance navigation. Posts were often emotionally charged and sometimes included humor, highlighting the ups and downs of family caregiving. It is critical to identify and address caregivers’ needs throughout their caregiving journey; our findings offer insights into the types of services and supports that should be more accessible, available, and affordable.

